# The *Ecology Underground* coalition: building a collaborative future of belowground ecology and ecologists

**DOI:** 10.1111/nph.17163

**Published:** 2021-02-17

**Authors:** Camille E. Defrenne, Elsa Abs, Amanda Longhi Cordeiro, Lee Dietterich, Moira Hough, Jennifer M. Jones, Stephanie N. Kivlin, Weile Chen, Daniela Cusack, André L. C. Franco, Albina Khasanova, Daniel Stover, Adriana L. Romero‐Olivares

**Affiliations:** ^1^ Climate Change Science Institute and Environmental Sciences Division Oak Ridge National Laboratory Oak Ridge TN 37830 USA; ^2^ Department of Ecology and Evolutionary Biology University of California Irvine 321 Steinhaus Irvine CA 92697 USA; ^3^ Department of Ecosystem Science and Sustainability Colorado State University Campus Delivery 1476 Fort Collins CO 80523 USA; ^4^ Department of Ecology & Evolutionary Biology University of Arizona Tucson AZ 85721 USA; ^5^ The Kellogg Biological Station Michigan State University Hickory Corners MI 48824 USA; ^6^ Department of Ecology and Evolutionary Biology University of Tennessee Knoxville TN 37996 USA; ^7^ College of Life Sciences Zhejiang University Hangzhou Zhejiang 310027 China; ^8^ Department of Biology Colorado State University Fort Collins CO 80523 USA; ^9^ Department of Integrative Biology University of Texas at Austin Austin TX 78712 USA; ^10^ US Department of Energy Washington DC 20585 USA; ^11^ Department of Biology New Mexico State University Las Cruces NM 88003 USA

**Keywords:** belowground ecology, diversity, equity and inclusion, fine roots, functional traits, microbes, process‐based models, spatial and temporal scales

## Introduction

Rising to meet global, interconnected challenges, such as food security and ecosystem resilience to global change, requires insights from across the Earth. This is especially true for belowground terrestrial ecological processes. Soil ecologists have answered this call: like mushrooms, large global datasets and databases on soil physics, chemistry and ecology are sprouting up everywhere (e.g. Fine‐Root Ecology Database (Iversen *et al*., 2018), Fun^Fun^ (Zanne *et al*., 2020), GlobalFungi (Větrovský *et al*., 2020), COntinuous SOil REspiration (Jian *et al*., 2020), Global distribution of earthworm diversity (Philips *et al*., 2019) etc.). It is now time to leverage synergies among these datasets to link the continuum of belowground processes to ecosystem functions (e.g. decomposition, nutrient cycling and soil respiration). Built on interdisciplinary and international collaborations, data synthesis that leads to a holistic understanding is one of the best ways of predicting the future of terrestrial ecosystems and for preserving ecosystem services under global change.

**Fig. 1:**
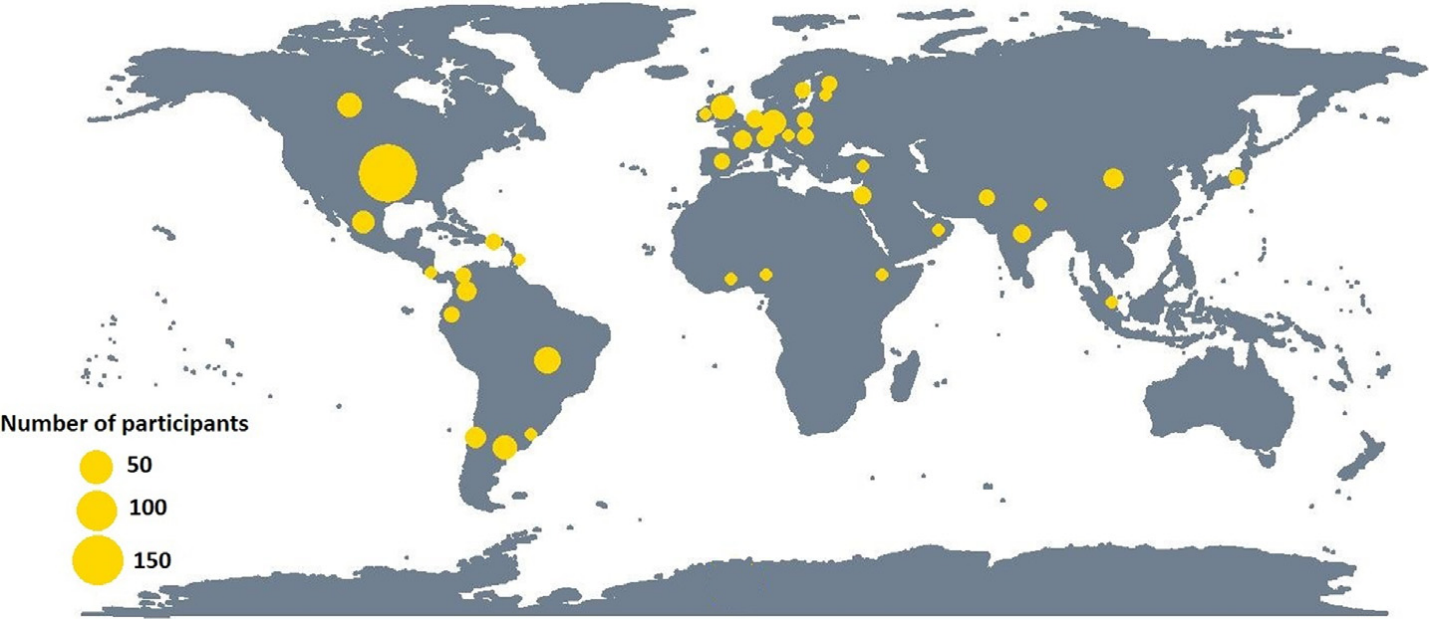
The online platform of *Ecology Underground* allowed for participation across the globe.

In 2020, global interconnectedness was on display as we saw a pandemic sweep across the world in weeks. The pandemic allowed the Ecological Society of America (ESA) to enter a new digital era by holding a fully virtual conference. While technology cannot yet replicate all aspects of an in‐person conference, the virtual platform increased accessibility and reduced the climate impact of the annual conference. It also provided an opportunity for early‐career plant and microbial ecologists from five ESA Organized Oral Sessions and one Inspire Session to organize *Ecology Underground*, a two‐day program of live virtual talks and open discussions on integrative belowground ecology on 4 and 5 August 2020. *Ecology Underground* was sponsored by *New Phytologist*, the ESA Soil Ecology Section, the US Department of Energy, Biological and Environmental Research program and the Oak Ridge Institute for Science and Education (ORISE), and gathered 453 participants over two days. The online platform allowed for participation across the globe (37 countries were represented; Fig. 1) and was an opportunity to think creatively about how scientific discussions and global collaborations can take place, now and in the future.

## Building an integrative future of belowground ecology

Dr Bala Chaudhary (DePaul University, IL, USA) opened *Ecology Underground* with her vision for the future of soil ecology and soil researchers (see her full talk here: https://vimeo.com/461592321). The discussion that followed her presentation focused on the importance of building comprehensive trait databases and of promoting Diversity, Equity and Inclusion (DEI) in STEM (Science, Technology, Engineering and Math). Building broad and inclusive communities not only increases geographic and organismal coverage of belowground ecological databases, but also creates synergies among diverse viewpoints, perspectives, and ways of thinking.

For the remainder of *Ecology Underground*, scientists from across the globe discussed: (1) linkages among climate and microbial community and ecosystem processes in light of rapid evolution, community assembly, trait‐based models and eco‐evolutionary dynamics; (2) integration of microbe‐ and plant‐focused studies to understand ecosystem responses to drought; (3) the availability of large soil datasets and how they can be applied to create mechanistic, process‐based ecosystem models; (4) new methods and conceptual frameworks in root trait research; (5) expanding root trait research to understudied areas (e.g. the tropics); and (6) broadening the root economic spectrum by taking into account fungal traits. The discussions from the six sessions of *Ecology Underground* highlighted three broad areas of synergy across these different topics.

## Synergy 1: the power of trait‐based approaches

'Wouldn’t it be amazing if we could predict belowground processes from drones and satellites aboveground by understanding linkages among leaf, wood, root, and microbial traits?' Colleen Iversen

**Fig. 2:**
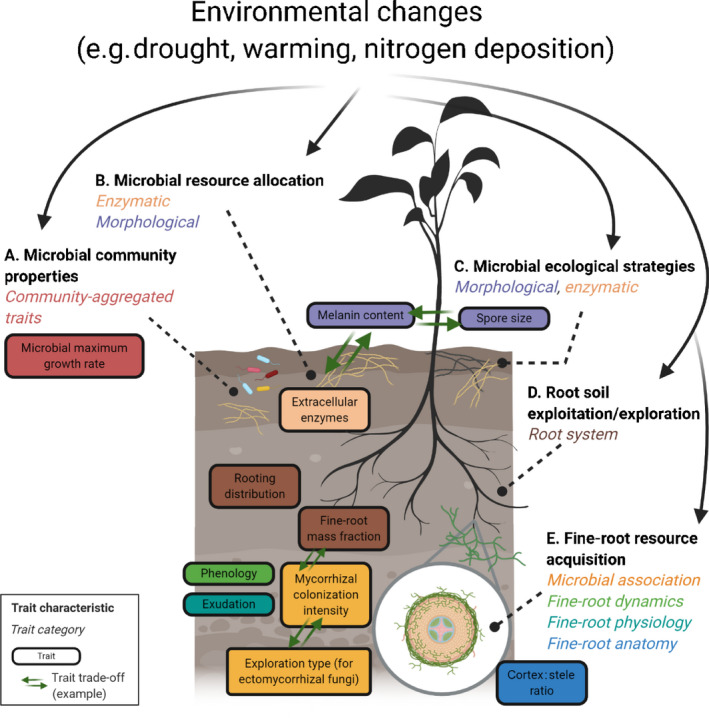
Diagram of key functional traits and trait trade‐offs of free‐living soil microbes, fine roots and mycorrhizal fungi discussed at *Ecology Underground*. These traits hold promise for assessing and modeling the responses of belowground processes to changing environments. Trait categories of free‐living soil microbes are from Hall *et al*. (2018) and Zanne *et al*. (2020), while fine‐root trait categories were extracted from Freschet *et al*. (2020b). Details on the trait characteristics can be found in the text. Created with BioRender (https://biorender.com/).

Trait‐based approaches are an alternative to species‐based approaches and place the emphasis on functional traits rather than species identities. This provides a framework for understanding how organisms affect ecosystem processes and community dynamics, by integrating functional information across species, space and time (Suding & Goldstein, 2008). *Ecology Underground* identified two overarching goals in applying trait‐based approaches to both plant roots and free‐living soil microbes: (1) identify critical functional traits driving ecosystem processes, and (2) identify trait trade‐offs that help to better model these processes, (Fig. 2). Within each of these goals there remain challenges specific to belowground trait‐based ecology, since lessons from aboveground trait‐based ecology and resource economics theory may not be directly translatable to plant roots and soil microbes due to competition between trade‐offs and environmental constraints.

### Identifying microbial functional traits

*Ecology Underground* identified three trait characteristics which hold promise for assessing and modeling responses of free‐living microbes to changing environments: (1) traits that can be predicted from community properties or community membership (Fig. 2; Hall *et al*., 2018); (2) traits that can cause changes in resource allocation in response to environmental changes and selective pressures, such as how experimental warming shifts fungal resource allocation from decay to cell metabolic maintenance (Romero‐Olivares *et al*., 2019); and (3) traits that underpin ecological guilds or strategies, such as cellulose decomposition or stress tolerance (Zanne *et al*., 2020). Phylogenetic signals or genome exploration for gene‐level traits can be helpful for identifying traits in organisms with no cultures and unknown function. Nonetheless, it is important to pursue phylogenetically independent approaches which have proven useful for the exploration of bacterial functional trait trade‐offs (e.g. evolutionary trade‐off between recalcitrant carbon use and the tolerance of low nitrogen conditions; Treseder *et al*., 2011).

### Identifying belowground plant functional traits

Extending fine‐root trait measurements from relatively‐easily measured morphological traits to include additional trait categories is key to a better understanding of belowground plant resource acquisition strategies and their relationship to ecosystem processes (Freschet *et al*., 2020b). Therefore, efforts should be made to investigate (4) fine‐root system traits, such as fine‐root mass fraction and rooting distribution (Fig. 2), and (5) traits that fall into multiple categories including fine‐root dynamics (e.g. phenology), physiology (e.g. exudation, enzymatic activity), biochemistry (e.g. phosphorus content), or anatomy (e.g. cortex : stele ratio) and traits associated with mycorrhizal fungi such as mycorrhizal type, mycorrhizal colonization intensity, the exploration type of ectomycorrhizal fungi, and hyphal branching.

### Relationships among traits

In order to mechanistically model plant and microbial mediation of global change on ecosystem processes, we must identify key relationships between plant and microbial traits. A first step is to estimate the associated costs of both plant and microbial traits and quantify the strength of their synergies and trade‐offs, such as those between stress resistance and resource acquisition (e.g. between osmolyte production for drought resistance and enzyme production for organic matter decomposition). Second, it is necessary to consider the nature of the environment (e.g. constant vs fluctuating; Rodríguez‐Verdugo *et al*., 2019) and biotic interactions (e.g. positive and negative interactions; Lee *et al*., 2019). In turn, relationships between fine‐root functional traits have proven useful for defining plant acquisition strategies belowground. Some key frontiers in this area of research include: (1) integrating root economics with root evolution (Valverde‐Barrantes *et al*., 2020); (2) identifying trade‐offs between fine‐root and mycorrhizal fungal traits and the emergent traits from their partnerships (McCormack & Iversen, 2019); (3) adopting a whole plant perspective (Weemstra *et al*., 2020); and (4) exploring patterns of intraspecific root and fungal trait variations (Defrenne *et al*., 2019). These frontiers will be crossed as we start linking root and fungal trait databases, create new databases (TraitAM, Chaudhary *et al*., 2020), develop novel techniques for the study of fine roots (Rhizovision, Seethepalli *et al*., 2020; EnRoot, Arnaud *et al*., 2019) and standardize root classification, sampling, processing and trait measurements (Freschet *et al*., 2020a).

## Synergy 2: mind the scale

‘We need big datasets to cross from microbiological process to macro‐ecological pattern.' Colin Averill

Integrating below‐ground processes into Earth system models and ecological forecasts requires a better understanding of the spatial and temporal consistency and turnover in microbial communities and fine‐root distribution and dynamics. Recently, the development of microbial habitat maps at the global scale (Stephanie Kivlin, University of Tennessee, USA) and the use of networks of observation sites revealed helpful ways to bridge the gap between belowground processes and macro‐ecological patterns. For instance, leveraging the National Ecological Observatory Network (NEON) enabled a better understanding of aerial arbuscular mycorrhizal fungal dispersal (Chaudhary *et al*., 2020) and the linking of rooting depth and soil nutrient distribution at the continental scale (Mingzhen Lu, Princeton University, NJ, USA). In turn, the use of the International Co‐operative Programme on Assessment and Monitoring of Air Pollution Effects on Forests (ICP Forests) made the exploration of microbial signatures at macro‐ecological scales possible because it enabled pairing observations of forest growth with those of forest microbiome composition and processes (nitrogen‐to‐carbon metabolism; Colin Averill, ETH Zürich, Switzerland).

Global biogeographical patterns appear to differ for soil invertebrates vs soil microbes, and we therefore need to examine them separately (Cameron *et al*., 2019). We also cannot assume that protecting aboveground organisms will be effective in protecting soil biodiversity since there is a substantial mismatch in terms of where species richness is highest for aboveground biodiversity and invertebrate soil biodiversity (Cameron *et al*., 2019). Furthermore, there are still large spatial and taxonomic gaps in our knowledge of soil invertebrate diversity, which introduces bias into macroecological studies (Guerra *et al*., 2020).

Accounting for temporal variation in fine‐root traits is crucial for a better understanding of belowground carbon allocation and root‐fungal resource acquisition strategies. For example, shifts in root production, mortality and/or phenology: (1) informed the belowground responses to warming and hurricanes (Daniela Yaffar, Oak Ridge National laboratory, TN, USA); (2) informed mangrove responses to reforestation (Marie Arnaud, University of Leeds, UK); and (3) significantly affected the total vegetation carbon simulated by the land model portion of E3SM (Energy Exascale Earth System Model; Daniel Ricciuto, Oak Ridge National Laboratory). However, we need better empirical data to identify tractable ways of modeling fine‐root phenology (Luke McCormack, Morton Arboretum, IL, USA).

Temporal scaling of microorganisms also seems to be more complex than originally estimated (Kivlin *et al*., 2020) and it requires studies which will examine responses in relation to climatic history and current variation. For instance, precipitation variation on decadal time frames can influence drought responses in plants and microbes (Knapp *et al*., 2020; Veach & Zeglin, 2020). Understanding how previous environmental conditions influence ecosystem responses to disturbance is critical in the design of experiments over relevant time scales to accurately predict future ecosystem responses to climate change. Another key challenge is to understand microbial evolution and adaptation rates in the field and to better link these data to data from laboratories. To achieve this goal, we need to study the physical and climatic conditions that microbes *truly* experience at the micro‐scale (Hall *et al*., 2018). Furthermore, little is known about the relative contributions of processes such as mutation and migration, or changes in the frequency of taxa or alleles that drive microbial trait variation. Efforts should be made to identify scales of variation (e.g. molecular, population, community) at which these processes operate and drive ecosystem processes (Alexander Chase, University of California, USA).

## Synergy 3: data integration into process‐based models

‘We have decent maps of the stars in the universe; it is about time we have decent maps of microbes in our own soils.' Stephanie Kivlin

**Fig. 3:**
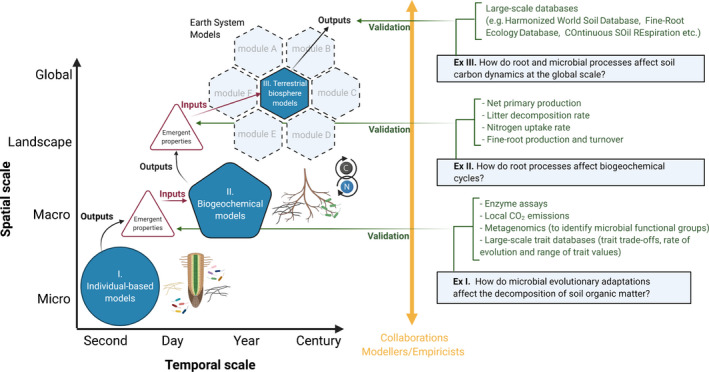
Conceptual diagram of process‐based models and the temporal and spatial scales at which they are used for predictions. Individual‐based models: (I) explicitly conceptualize individual microbes or a single plant root and involve a high level of complexity in the description of microbial functional groups (*c*. 100 groups) and molecular dynamics (e.g. extracellular enzymes, osmolytes, organic polymers, etc.). In biogeochemical models (II), soil microbes are grouped by functional types (e.g. fungi and bacteria) and root distribution can be coupled with highly resolved cycling of water and nutrients within the plant–soil system. Terrestrial biosphere models (III) are modules of Earth System Models; some do not explicitly represent soil microbes, and root parameters are often fixed within and across plant functional types. Informing model input values and validating model outputs (i.e. emergent properties) requires strong collaborations among modelers and empiricists. The question of interest (see Ex I, II and III) determines the type of model used which, in turn, determines the type of empirical data (green text) needed to validate model outputs. Eventually, emergent properties of models operating at lower scales may become inputs or inform the conceptualization of model processes for models operating at higher scales. Created with BioRender (https://biorender.com/).

Meeting the grand challenge of improving the conceptualization of microbes and plant roots in terrestrial biosphere models (modules of Earth System Models; Fig. 3, Ex III) requires that we generate trait‐based ecological information belowground. This information is essential to inform inputs of models operating at different spatial and temporal scales. Furthermore, the use of models which represent multiple scales is critical to bridge the gap between soil ecological observations at locations across the globe and biosphere model predictions.

Model selection depends on the question of interest (Fig. 3, Ex I–III) and determines the type of empirical data needed for model parameterization and validation. For example, individually based models can be used to test the outcome of interactions between microbial taxa (Allison *et al*., 2010), the effect of evolving microbial strains on local decomposition (Fig. 3, Ex I; Abs *et al*., 2020), or drought legacy in soil microbiomes (Steven Allison and Bin Wang, University of California, USA ). Biogeochemical models focus on highly‐resolved biogeochemical cycling within an ecosystem and are therefore useful to investigate processes related to changes in soil carbon in response to environmental changes, for example by integrating microbial functional groups (MIcrobial‐MIneral Carbon Stabilization (MIMICS) model; Wieder *et al*., 2015) or the protection of soil organic matter (Sulman *et al*., 2014). Eventually, emergent properties of biogeochemical models such as the decomposition rates of microbial functional groups may become inputs for terrestrial biosphere models, in which microbes are not explicitly represented (yet) and where fine‐root traits (e.g. rooting depth distribution, carbon‐to‐nitrogen ratio) have fixed parameters across plant functional types (Iversen *et al*., 2017).

Informing model input values and validating model outputs requires strong collaborations between modelers and empiricists. Modelers using individual‐based models need empiricists to provide them with a simple framework for microbial traits, by, for example, linking microbial diversity and ecosystem processes (e.g. Mark Anthony and Colin Averill, ETH Zürich, Switzerland ) or connecting descriptive diversity patterns and community traits to community level functions (e.g. Romero‐Olivares *et al*., 2019). Modelers also need empiricists to (1) pair measurements of interest with those that models require; for example, effort should be made to pair measurements of fine‐root morphology and chemistry with fine‐root function, and (2) provide them with microbial trait trade‐offs or range limits for a given trait (Abs *et al*., 2020). Empirical data such as local carbon dioxide emissions or community‐level microbial biomass can be important to validate the emergent properties of individual‐based models while measurements such as ecosystem‐level net primary production or soil decomposition rates over time in long‐term warming experiments (Melillo *et al*., 2017) or nitrogen addition experiments (Morrison *et al*., 2018), can be needed to validate biogeochemical model outputs. Lastly, terrestrial biosphere models require large scale data (e.g. the Harmonized World Soil Database, FAO, 2012) and/or emergent properties from biogeochemical models for both model parameterization and model evaluation or validation. However, caution should be used when relying on inverse modeling (i.e. tuning models to match large scale data) because important fine‐scale mechanisms may be overlooked.

## Building an inclusive future of belowground ecologists

‘We cannot perform global ecology without embracing the diversity of scientists around the globe.' Bala Chaudhary

The events that occurred in May and June 2020 in the USA and around the world (e.g. the murder of George Floyd, Black Lives Matter marches and demonstrations, #BlackInTheIvory, #ShutDownSTEM, #ShutDownAcademia, etc.) called new attention to the need for anti‐racism in STEM. Recent efforts towards promoting Black, Indigenous, and People of Color (BIPOC) in STEM include: (1) encouraging the scientific community to build anti‐racist labs (Chaudhary & Berhe, 2020); (2) promoting Black excellence in ecology and evolution (Schell *et al*., 2020); (3) helping BIPOC students to engage in conservation science (Doris Duke Conservation Scholars Program at the University of Washington); and (4) supporting scientists who self‐identify as women of color and work in ecology, evolutionary biology or allied fields (e.g. WOCinEEB slack online community, created by Bala Chaudhary, wocineeb@gmail.com; or Halsey *et al*., 2020). These efforts are necessary because BIPOC are underrepresented in environmental fields worldwide (e.g. Taylor, 2015) despite demonstrating high environmental concern and having strong motivations such as achieving ecological and social well‐being in their communities (Tania Schusler, Loyola University of Chicago, IL, USA).

Science and equity are interdependent – research shows more diverse groups produce more novel ideas (Hofstra *et al*., 2020), therefore, scientific communities should be diverse at all levels (e.g. students, early‐career researchers, faculty members). In addition, actions promoting DEI should primarily be carried out by people in power such as faculty members and leaders, and the onus should not be on graduate students or BIPOC themselves, although their contributions should be welcomed and valued. To value the scientific expertise and important perspectives of PEERs’ (Persons Excluded because of their Ethnicity or Race), departments must actively support them speaking about their science as well as their experiences navigating STEM and support them serving on both scientific and diversity committees. In order to increase DEI in STEM, one can create new programs that integrate science and DEI or create new funding opportunities that specifically require applicants to integrate science and DEI. Furthermore, organizations should be intentional about recruiting BIPOC students and faculty members instead of simply removing ‘roadblocks’ such as application fees. Similarly, they must be intentional about supporting the people they recruit such as taking steps to create communities of BIPOC, and hiring BIPOC in clusters to avoid feelings of isolation and exclusion in white‐dominated spaces.

Lastly, future events would be greatly improved if organizers: (1) have conversations about DEI early and throughout the process to ensure that the team has a shared knowledge and vision, and can act on that vision throughout the organizing process; (2) include PEERs co‐organizers and speakers as this would help create synergies among ways of thinking (e.g. Hagan *et al*., 2020); and (3) encourage organizations to advertise, including scientific societies focused on representing PEERs in STEM (e.g. Society for Advancement of Chicanos/Hispanics & Native Americans in Science – SACNAS).

## Conclusions

In a world of social distancing to slow the spread of a global pandemic, collaborative and critical scientific conversations can still happen via online meetings. Through two days of online synchronous presentations and discussion that leveraged sessions proposed within the ESA framework, *Ecology Underground* identified three critical areas of belowground ecology that need further research: (1) the use of trait‐based approaches for linking plants, microbes, and ecosystem processes; (2) the identification of relevant spatial and temporal scales for studies in belowground ecology; and (3) the development of models that connect microscale dynamics to predict belowground processes globally. Advancement of these research areas will require strong global networks, cross‐disciplinary collaboration, and a diversity of perspectives only achievable through a diverse community of ecologists. Creating such a community will require active work from people at all levels (students to tenured faculty members) to recruit, support and promote BIPOC and other historically excluded people in science. As ecologists, we know that diversity is essential to building resilient ecological communities. Applying the same principles to our community of scientists will surely lead us to new insights, better science, and a more vibrant research community.
